# Practice of Family Physicians in the Management of Osteoporosis Among Older Female Patients at King Abdulaziz Medical City, Riyadh, Saudi Arabia

**DOI:** 10.7759/cureus.22387

**Published:** 2022-02-19

**Authors:** Siham M AlAnazi, Amani A AlKhamali, Mohammed A AlAteeq

**Affiliations:** 1 Family Medicine, King Abdulaziz Medical City, Riyadh, SAU; 2 Family Medicine, Ministry of National Guard - Health Affairs, Riyadh, SAU; 3 Family Medicine, Ministry of National Guard - Health Affairs, King Abdullah International Medical Research Center, King Saud Bin Abdulaziz University for Health Sciences, Riyadh, SAU

**Keywords:** pharmacotherapy, women health, elderly, bone health, general practice

## Abstract

Background

Osteoporosis is one of the major worldwide health concerns and carries medical, social, and financial impacts due to its significant morbidity and mortality. The aim of this study was to explore the practice of family physicians in osteoporosis management in older female patients.

Method

This study was performed in a cross-sectional manner. Data, including patient and disease characteristics and management practices, were obtained from the electronic patient chart for female patients aged 60 years and older who were followed in family medicine clinics at King Abdulaziz Medical City, Riyadh, Saudi Arabia, during the period from January 1, 2018, to December 31, 2020. Management was checked and evaluated against osteoporosis management guidelines.

Results

In 42 patients out of 250, an osteoporosis diagnosis was missed. Among the 208 charts that were reviewed, the mean patient age was 72.06 years, and the mean duration of the disease according to the initial dual-energy X-ray absorptiometry (DXA) scan was 3.68 years. Laboratory tests were performed for only about half of the patients. Vitamin D was given only to 54.3% while calcium supplements were given only to 53.4%. Almost one-third of the patients have not been prescribed any antiosteoporosis medications.

Conclusion

The current study documented that older females with osteoporosis were not well-managed in the investigated family medicine settings. Continuous professional development activities and periodic practice reviews are suggested to improve this situation.

## Introduction

Osteoporosis is “a skeletal disorder characterized by compromised bone strength predisposing to an increased risk of fracture” [1].

Osteoporosis is one of the significant global health concerns with 200 million cases reported worldwide [2]. After heart disease, osteoporosis is considered the most important health issue in developed countries [3] associated with high rates of morbidity and mortality with marked social and financial impact [4].

Despite the availability of updated guidelines for the management of osteoporosis from well-known organizations, many studies worldwide have reported a lack of appropriate management of osteoporosis patients and those with fragility fractures worldwide [5]. A study done in the United States (US) reported that among primary care physicians, the rate of those who reported using bone turnover markers for screening, diagnoses, or monitoring osteoporosis in 1998 was only 19%; this rate tripled to 55% by 2006. According to study findings, only 15% of patients were prescribed bisphosphonates, which had increased to 59% by 2006 [6].

Another retrospective cohort study done in Canada reported that the intervention rate among primary physicians in the treatment of osteoporosis is generally low. The study found that in 2000, among women aged 65 and above, only 11% used osteoporosis medication, and by 2009, the rate increased to only 27% [7]. Likewise, in 2014, a study was conducted in the Czech Republic and found that only 60% of general practitioners were following the osteoporosis guidelines [8]. In Australia, a one-day audit done for all admitted osteoporosis patients found that only 40% of them were taking antiosteoporosis therapy, and among those with a fracture or history of fracture, only 31% received antiosteoporosis therapy and only 16% of those had follow-up visits [9]. 

Locally, a study done with patients who were admitted with a fragility fracture showed that bone mineral density (BMD) testing was ordered in only 24.1%. It was also reported that vitamin D was ordered for 14.9% while 37.6% of patients were given vitamin D and calcium. It was also found that about 60.8% of patients did not receive any treatment for osteoporosis [10]. A more recent local study reported that among patients with fragility fractures, 64.1% had not received appropriate treatment at the time of discharge and after three months of follow-up, this rate was reduced to 32.9%, which also decreased further after one year to 25.74% [4].

The aim of this study was to explore the practice of family physicians in the management of osteoporosis among older female patients.

## Materials and methods

Study type and setting

This study was a cross-sectional, descriptive, and analytical chart review study. It included patients who were followed at three primary care clinics at King Abdulaziz Medical City, Riyadh, Saudi Arabia: (1) King Abdulaziz Housing Clinics (Iskan), (2) National Guard Comprehensive Specialized Clinic (NGCSC), and (3) Health Care Specialty Center (HCSC). All three centers provide health care for acute and chronic conditions for all National Guard employees and their dependents and have walk-in and booking appointment systems that are supported by both curative and preventive health services.

Study subjects

All female patients, 60 years old and above, with abnormal DXA scans consistent with osteoporosis according to the WHO criteria [5,7], from Jan 1, 2018, to December 31, 2020, were identified and included. Patients with the following conditions causing secondary osteoporosis were excluded: type 1 diabetes mellitus, hyperthyroidism. hyperparathyroidism, growth hormone deficiency, acromegaly, hypopituitarism, inflammatory bowel disease, celiac disease, gastric bypass surgery, hemochromatosis, chronic liver diseases, multiple myeloma, systemic mastocytosis, beta-thalassemia major, connective tissues disease, autoimmune disorders, and corticosteroids users.

Study variables

Study variables were identified to meet study objectives. The variables were reviewed by two experts, a family consultant and an endocrine consultant specializing in osteoporosis. Study variables included several parameters: (1) patient-related information (age, body mass index [BMI], disease duration in months according to the first abnormal DXA, chronic medical conditions, documented history of fractures), (2) management-related (request for laboratory studies [vitamin D, calcium, thyroid-stimulating hormone, alkaline phosphatase, creatinine, and phosphate levels]), (3) initial and serial DXA, (4) prescription of vitamin D, calcium, and antiosteoporosis medications, and (5) follow-up.

The follow-up variable was divided into three categories: (1) one to four years, (2) five to nine years, and (3) 10 years or older. The T-score variable in the initial BMD results was divided into three categories: (1) −4.00 to −5.10, (2) −3.00 to −3.90, and (3) −2.50 to −2.90. With respect to disease duration, it was also divided into three categories: (1) < 2 years, (2) 2-5 years, and (3) > 5 years.

Data collection

Patients' medical records were retrospectively identified and reviewed. Data were obtained from electronic charts at BestCare. Family physician management of the condition was checked and evaluated against the guidelines. Documented management of the patient during the first visit after the initial DXA was reviewed and used to judge the physician’s practice. If laboratory results were already available within three months or less prior to the first visit, laboratory tests were considered done.

Statistical analysis

Data were analyzed using Statistical Package for the Social Sciences (SPSS), version 23 (IBM Corp., Armonk, NY). Quantitative variables were reported in the form of mean and standard deviation. For qualitative variables, it was in the form of frequency and percentages.

Ethical approval

Ethical approval was obtained from the Institutional Review Board at King Abdullah International Medical Research Center, Riyadh, Saudi Arabia, study number RC20/043/R on March 11, 2020. Data collection sheets were coded using three‑digit serial numbers and were maintained by the co‑investigator. Participants could not be identified after the collection of the datasheets. The study was carried out according to the principles of the Helsinki Declaration.

## Results

A total of 250 medical records with BMD-based osteoporosis were found, out of which 42 were missed by physicians (for example, BMD results were not seen). The review and analysis were done for the remaining 208 patients.

As shown in Table 1, the patient’s mean age was 72.06 ± 7.82 years. The largest age group was between 60 and 70 years (50.7%) followed by the age group from 71 to 80 years (37.2%) while 12.1% were aged 80 years and above. The mean BMI was 30.88 ± 6.58. About half of patients (44.71%) were obese followed by overweight (30.2%), normal (15.38%), morbidly obese (8.17%), and underweight (1.44%). Regarding medical history, most patients (99%) suffered from chronic diseases. Table 2 represents different chronic medical conditions with frequencies.

**Table 1:** Participants’ related variables (n = 208)

		No.	%	Mean ± standard deviation (SD)
Age groups	60–70	105	50.5	72.06 ±7.82
	71–80	78	37.5	
	Over 80 years	25	12.00	
Body Mass Index	underweight	3	1.44	30.88 ± 6.58
	normal	32	15.38	
	overweight	63	30.2	
	obesity	93	44.71	
	morbid obesity	17	8.17	
Chronic diseases	no	2	1.0	-
	yes	206	99.0	

**Table 2:** Chronic diseases among study subjects

	No.	%
Type 2 diabetes mellitus	117	56.3
Hypertension	126	60.6
Chronic kidney disease	6	2.9
Hypothyroidism	39	18.8
Osteoarthritis	124	59.6
Obesity	110	52.88

Table 3 presents disease-related variables. Concerning disease duration, the mean was 3.68 ±3.48 years. Less than half (42.5%) of the patients had the disease for two years or less, 34.5% had the disease for two to five years, and 23% had the disease for more than five years. The mean result of the T-score in the initial BMD scans was −3.05 ± 0.51. Fragility fractures were reported in 13.5% of patients.

**Table 3:** Disease-related variables DXA: dual-energy X-ray absorptiometry

		No.	%	Mean ± S.D
Disease duration (n = 200)	< 2 years	85	42.5	3.68 ± 3.48
	2–5 years	69	34.5	
	> 5 years	46	23.0	
Initial DXA results (T-score) (n = 208)	−4.00–(−)5.10	13	6.26	−3.05 ± 0.51
	−3.00–(−)3.90	84	40.38	
	−2.50–(−)2.90	111	53.36	
Fragility fracture (n = 208)	no	180	86.5	-
	yes	28	13.5	

Concerning the management practice (Table 4), vitamin D levels were requested for 42.8% of patients, calcium for 52.4%, thyroid-stimulating hormone (TSH) for 51.4%, alkaline phosphatase for 48.1%, creatinine for 66.8%, and phosphate for 51.4%. Fifty-seven patients (27.4%) had a second DXA scan done with a mean of −3.10 ± 0.60, and only 3.4% underwent a third DXA scan with a mean of −2.74 ± 0.51. Almost half of the patients were only prescribed vitamin D and calcium. Regarding medications, 73.1% of patients were prescribed bisphosphonates while 26.9% were not given any antiosteoporosis medications. Concerning follow-up, 36.1% had no follow-up visits, 32.7% had been followed up one to four times, 20.7% had been followed up five to nine times and 10.6% had been followed up 10 or more times. In addition, results indicated that 11.1% of patients were referred to either orthopedic or endocrine clinics.

**Table 4:** Management practice DXA: dual-energy X-ray absorptiometry

Variable		No.	%
Requested laboratory	Vitamin D	89	42.8
	Calcium	109	52.4
	Thyroid-stimulating hormone (TSH)	107	51.4
	Alkaline phosphatase	100	48.1
	Creatinine	139	66.8
	Phosphate	107	51.4
Prescribed treatment	Vitamin D	113	54.3
	Calcium	111	53.4
	Bisphosphonates	152	73.1
Repeated dual-energy X-ray absorptiometry (DXA) scans	Second DXA scan	57	27.4
	Third DXA scan	7	3.4
Number of follow-up (FU) visits	None	75	36.1
	1–4 times	68	32.7
	5–9 times	43	20.7
	≥10 times	22	10.6

## Discussion

In this study, we aimed to explore the management practice of osteoporosis in a family medicine setting against current consensus guidelines.

Most patients in this study (83%) were either overweight (30%) or obese (53%). This finding is higher than what is reported nationally for overweight and obesity among adult Saudi females [11]. Conventionally, a high BMI is considered a protective factor against osteoporosis [12], but recent studies are challenging this and proposing that weight gain may enhance bone loss. In addition, obesity-related chronic conditions, such as diabetes mellitus, hypertension, and dyslipidemia, have a negative impact on bone health [13-14].

With respect to osteoporosis severity, according to the WHO classification, any BMD of T-score < −2.5 with a fragility fracture is considered severe osteoporosis [3]. In the current study, we reported 28 patients (13.5%) with fragility fractures. This finding is less than what is reported in 2012 by Sadat-Ali et al. in their epidemiological analysis in which the vertebral fracture prevalence was reported between 20% and 24% [15]. International figures, for example, in Italy and China, have reported a higher prevalence of fragility fractures [16-17]. It should be noted that vertebral fractures, which account for about 50% of osteoporosis-related fractures, are mostly asymptomatic and found incidentally with imaging of the lumbosacral spine [18]. Therefore, in this study, fragility fractures might have been underreported.

Disease duration was reported in the current study based on the date of the initial DXA scan. Our results showed that 42.5% of patients had their initial DXA less than two years before the study started. Considering the mean age of patients in this study, which was 72.06 years, this finding means that most of the patients were not screened for osteoporosis at the proper time.

In reviewing management practice, apparent deficiencies with variability in different domains were found, either in the assessment of patients or prescribing pharmacotherapy. Almost half of the patients did not have the basic laboratory investigations for osteoporosis. For example, only 42.8% and 52.45 of patients had been tested for vitamin D and calcium levels, respectively. 

Optimizing vitamin D and calcium levels in osteoporosis patients is paramount to the management of osteoporosis. Nevertheless, almost half of the patients in this study did not receive vitamin D and calcium supplements, and almost one-third were not prescribed any antiosteoporosis medications.

These results are consistent with multiple previous reported studies, which showed many deficiencies and gaps in the assessment and management of osteoporosis [6-7,19-24].

For example, locally, Sadat‑Ali et al. reported even a smaller number of patients who were tested for vitamin D levels [8]. Similarly, another hospital-based study by Sadat-Ali et al. reported that only 42.5% of patients were prescribed vitamin D and calcium, and only 9.6% were given bisphosphonate treatment at discharge after management for femur fragility fractures [4].

In reviewing the practices of American primary care physicians in the management of osteoporosis, Stephen et al. reported that only 36% of osteoporosis patients were offered antiresorptive agents and calcium and vitamin D [19]. In a recent study done across Europe for 3798 postmenopausal women from eight European countries, there was a large treatment gap in women aged ≥ 70 years at increased risk of a fragility fracture in routine primary care [22]. Moreover, a study done in Australia revealed that 72% of patients were not prescribed any osteoporosis medications [25].

In the Czech Republic, 60% of 540 interviewed primary care physicians reported adherence to osteoporosis management guidelines. However, they listed several limitations in the management of osteoporosis mainly being unauthorized to prescribe drugs, financial limits by insurance companies, patient nonadherence, and prioritized health problems [8].

Limitations

One limitation is the retrospective nature of the study; hence, data may be affected by the documentation of managing physicians and may not accurately reflect real-world practice in the domains of education, advice, and follow-up. However, this effect does not pertain to doctors’ orders, either investigation or medication-related, which are entered in the electronic system. Another limitation is the relatively small number of patients.

## Conclusions

Although osteoporosis is a well-known disease because of its complications and significant morbidity and mortality, the current study documents improper management of the condition at the level of family medicine practice. More should be done in terms of continuous professional development activities and regular auditing of the practice to improve this situation.
